# A novel pharmacological strategy using nanoparticles with glutathione and virgin coconut oil to treat gentamicin-induced acute renal failure in rats

**DOI:** 10.1007/s00210-024-03303-4

**Published:** 2024-08-02

**Authors:** Mahmoud S. Sabra, Essmat A. H. Allam, Mohamed Abd El-Aal, Nessma H. Hassan, Al-Hassan Mohammed Mostafa, Ahmed A. N. Ahmed

**Affiliations:** 1 Pharmacology Department, Faculty of Veterinary Medicine, Assiut University, Assiut, 71526 Egypt; 2 Department of Pharmacology and Toxicology, Faculty of Pharmacy, Assiut University, Assiut, 71526 Egypt; 3 Chemistry Department, Faculty of Science, Assiut University, Assiut, 71516 Egypt; 4 Department of Pathology and Clinical Pathology, Agricultural Research Centre, Animal Health Research Institute, Assiut, 71526 Egypt; 5 Pharmacology Department, Faculty of Medicine, Al-Azhar University, Assiut Branch, , Assiut, 71526 Egypt

**Keywords:** Gentamicin, ARF, Nanoparticles, GSH, NGAL, KIM-1

## Abstract

In acute renal failure (ARF), the glomerular filtration rate is reduced, and nitrogenous waste products accumulate persistently, which can last anywhere from a few hours to several days. There is hope for a reversal of the rapid loss of renal function caused by this condition. This study, with gentamicin-induced acute ARF as a prospective setting, sets out to examine the reno-protective benefits of virgin coconut oil (VCO) and GSH. Furthermore, the study evaluated the effect of medication nanoparticle compositions on several kidney function markers. The induction of ARF is achieved with the intraperitoneal injection of gentamicin. To assess renal function, rats underwent 24 h of dehydration and hunger before their deaths. The study examined various aspects, including kidney function tests, markers of oxidative stress, histology of kidney tissue, inflammatory cytokines, immunohistochemistry expression of nuclear factor-kappa B (NF-κB), and specific biomarkers for kidney tissue damage, such as kidney injury molecule-1 (KIM-1) and neutrophil gelatinase–associated lipocalin (NGAL). The results of our study indicated that the combination of VCO and GSH, using both regular and nanoparticle formulations, had a better protective impact on the kidneys compared to using either drug alone. The recovery of renal tissue and serum markers, which are symptomatic of organ damage, indicates improvement. This was also demonstrated by the reduction in tubular expression of TNF-α, IL-1β, KIM-1, and NGAL. The immunohistochemical studies showed that the combination therapy, especially with the nanoforms, greatly improved the damaged cellular changes in the kidneys, as shown by higher levels of NF-κB. The study shows that VCO and GSH, when administered individually or combined, significantly improve ARF in a gentamicin-induced rat model, highlighting potential therapeutic implications. Notably, the combined nanoparticulate formulations exhibit substantial effectiveness.

**Supplementary Information:**

The online version contains supplementary material available at 10.1007/s00210-024-03303-4.

## Introduction

Approximately 50–60% of severely ill people and 5–7.5% of hospitalized patients suffer from ARF. Researchers have observed ARF in over half of patients receiving critical care (Hoste et al. 2015). These days, a rapid reduction in urine production or an abrupt rise in serum creatinine levels over a certain time period might diagnosis acute renal failure (Yoon et al. 2022). Treatment options for patients with ARF are conditional on the available resources and the nature of the therapeutic context. The effectiveness of many commonly used treatments is still up for discussion, but there is mounting evidence that many interventions, especially when given at the same time, might have a positive impact. While there is still much debate, there is agreement on many ARF management treatments and techniques, which opens the door to useful quality control and benchmarking (Ronco et al. 2019).

A common antibiotic for gram-negative bacteria is gentamicin, an aminoglycoside. The compound’s nephrotoxic properties limit its therapeutic usefulness. In gentamicin-induced ARF, the histological changes include glomerular atrophy, tubular necrosis, tubular fibrosis, and inflammation. There are also higher levels of urea nitrogen, creatinine, and non-protein nitrogen in the blood (Mahi-Birjand et al. 2020). Gentamicin can reduce renal structure and function by increasing the production of reactive oxygen species (ROS) in the renal cortex, according to research. These species include superoxide anions, hydroxyl radicals, hydrogen peroxides, and reactive nitrogen species (Gamaan et al. 2023). Through its effects on mitochondria, gentamicin triggers the intrinsic process of cell death. At the molecular level, caspase activation and other chemicals generated by mitochondria regulate apoptosis (Babaeenezhad et al. 2021).

Evidence suggests that VCO has antioxidant and anti-inflammatory properties (Famurewa et al. 2019). The edible part of the coconut fruit (*Cocos nucifera* L.) naturally extracts VCO without the need for chemical bleaching or refining (Jaarin et al. 2014). Because the extraction process uses little to no heat and no chemicals, VCO is more advantageous due to its approach. This means more of the beneficial natural components, such polyphenols, which boost antioxidant defenses, stay in the final product (Nevin and Rajamohan 2006).

The nutritional and medicinal benefits of coconut oil (CO) have been well-documented for quite some time. Studies have demonstrated that VCO can decrease oxidative stress by decreasing lipid peroxidation, cleaning up free radicals, and strengthening the antioxidant defense system (Iranloye 2013). VCO enhances memory while simultaneously reducing oxidative stress and diseases (Dosumu et al. 2012; Rahim et al. 2017). VCO appears to have antioxidant and anti-inflammatory characteristics that protect against the tissue toxicity of anti-neoplastic and anti-retroviral treatment (Famurewa et al. 2019; Ogedengbe et al. 2018). It has also been shown to increase weight reduction and improve thyroid function (Takeuchi et al. 2008).

In addition to its anti-inflammatory and anti-ulcerogenic effects, CO may raise HDL cholesterol levels while lowering LDL cholesterol levels in blood and tissues, according to another research (Anosike and Obidoa 2010; Nevin and Rajamohan 2006). Furthermore, the data suggested that VCO may be beneficial in preventing diclofenac-induced nephrotoxic damage and reducing renal damage in diabetics (Akinnuga et al. 2014; Alatawi and Alshubaily 2021; Famurewa et al. 2020). A recent study suggests that curcumin-loaded CO microemulsion represents a successful formulation for neurodegeneration treatment (Patil et al. 2022). Furthermore, VCO-encapsulated NPs are a powerful nanoscale antibiotic against multidrug-resistant bacteria (Talib et al. 2021).

Protecting cells from oxidative damage and keeping cellular redox processes in equilibrium are both dependent on reduced glutathione (GSH) synthesis (Raj Rai et al. 2021). GSH synthesis dysregulation is observed in various medical circumstances such as renal failure (Adedapo et al. 2021), liver injury (Lu 2020), diabetes, neurological diseases (Aoyama 2021), organ fibrosis (Mansour et al. 2020), and cardiovascular disease (Matuz-Mares et al. 2021). The treatment and management of kidney disease has become a major global burden. The onus is now on researchers to find a way to enhance renal treatment without causing unwanted side effects while yet delivering the medicine to the site of action. Renal issues are now being treated with an increasing array of nano-based medication delivery technologies (Merlin and Li 2022).

In the development of nanoscale sensing technologies, iron oxide nanoparticles (Fe_3_O_4_ -NPs) are being looked at as a possible alternative to gadolinium-based magnetic resonance imaging contrast agents, which are harmful to the kidneys. Fe_3_O_4_ particles are used in functional imaging for quantitative perfusion, quantitative glomerular filtration rate, and tubular function assessment, as well as cellular imaging for intrarenal phagocytosis in inflammatory nephropathy (Ma et al. 2020). Chitosan (CS) is a biocompatible and degradable polymer that can be targeted especially to the kidneys (He et al. 2012; Sabra et al. 2024a). The research has explored the possibility of CS NPs as drug delivery vehicles for oral administration in renal illness (Wang et al. 2021).

As a result, the goal of this study is to investigate the potential effects of both traditional and NP formulations of VCO extract and GSH against gentamicin-induced nephrotoxicity in rats.

## Materials and methods

### Animals and the induction of ARF

The Institutional Animal Care & Ethics Committee of the Faculty of Veterinary Medicine at Assiut University granted consent to this experiment (approval number: 06/2024/0187), and all ARRIVE regulations were followed. The adult male albino rats used in the study weighed 150–200 g, were 8–10 weeks old, and were part of the investigation. The veterinary medicine faculty at Assiut University maintained a controlled environment for the animals at their animal facility, where they were provided with food, water, and standard laboratory lighting and darkness patterns.

Intraperitoneal injections of 100-mg/kg gentamicin were administered to rats for 7 days to produce acute renal failure. Blood samples were collected at the conclusion of the study, and serum was extracted from them. The next step before euthanasia was to breathe 5% isoflurane to render the rats fully unconscious. When rats did not respond to stimulation of the head and limbs, cervical dislocation was used to quickly kill them. After 10 s of having their necks dislocated, rats were deemed dead if they stopped breathing and did not react to systemic stimulation. Samples of kidneys were homogenized and stored at − 80 °C after tissue removal. Histopathological and immunohistochemical examinations required the preservation of kidney tissues in 10% phosphate-buffered formalin.

### Experimental animal grouping

There were a total of nine groups of animals, with six rats in each. A week was the length of the trial period. In keeping with prior research, the rats were given VCO orally at a dose of 15 ml/kg p.o. (Al-Joufi et al. 2022; Mu’izuddin et al. 2018). A 100-mg/kg intraperitoneal dose of GSH was given to the rats in accordance with the prior research (Babaeenezhad and Dezfoulian 2023). Medications were administered to all groups for 7 days after the acute renal failure model was created. Group 1 served as a control group that received just saline as an experimental intervention. The second group, designated as ARF, was given gentamicin to cause ARF. Gentamicin and VCO (15 ml/kg p.o.) were administered for 7 days to group 3. For 7 days, members of group 4 were given gentamicin and GSH (100 mg/kg i.p.). A combination of GSH (100 mg/kg i.p.), VCO (15 ml/kg p.o.) and gentamicin were administered to group 5 for 7 days. For 7 days, members of group 6 were given gentamicin and VCO (15 ml/kg p.o.)-iron oxide (Fe_3_O_4_) NPs. Gentamicin and GSH (100 mg/kg i.p.)-CS–laden NPs were administered to group 7 for a duration of 7 days. For 7 days, members of group 8 took gentamicin in addition to GSH-CS NPs, as well as a mixture of VCO-Fe_3_O_4_ NPs. Animals in group 9 were exposed to gentamicin-induced ARF for 7 days using Fe_3_O_4_ and CS-based NPs as carriers.

### Materials

The following substances were utilized as received, without undergoing additional purification: anhydrous ferric chloride (FeCl_3_), ammonium ferrous sulfate heptahydrate (NH_4_)_2_Fe(SO_4_)_2_·6H_2_O), and sodium hydroxide (NaOH) were obtained from Alpha chemicals (Cairo, Egypt). Chitosan (CS, low molecular weight, Sigma-Aldrich, USA), sodium tripolyphosphate (TPP, Na_5_P_3_O_10_, assay: 94%, Qualikems, India), GSH (C_10_H_17_N_3_O_6_S, assay: 95%, AKSCI, USA), and acetic acid (C_2_H_4_O_2_, assay: 99%, Alpha Chemicals, Cairo, Egypt).

### Virgin coconut oil extraction procedure

The VCO was isolated in accordance with the prior research of Famurewa et al. (2020). The Assiut Town market in Egypt was the source for the mature coconuts, certified by an authority in the field of medicinal plants at Egypt’s Assiut University’s Faculty of Agriculture. After removing the meat from the coconut using a knife, it was chopped into small pieces and ground into a thick slurry with 400 ml of distilled water. The result was a milky filtrate that was let to sit for 48 h after being passed through a cheesecloth sieve. The upper layer was delicately separated and then heated to a gentle 50 °C in order to extract the oil that had separated. To prepare it for usage, the new VCO was progressively spoon-withdrawn and filtered into a container that could be sealed tightly.

### Synthesis of *iron* oxide (Fe_3_O_4_) and *iron* oxide-virgin coconut oil (Fe_3_O_4_-VCO) nanocomposite

Iron oxide NPs were synthesized using the co-precipitation method as described in the literature (Abd El-Aal et al. 2023; Aldosari et al. 2023; Najjar et al. 2022). To prepare the solution (A), anhydrous FeCl_3_ (21 g) and (NH_4_)_2_Fe(SO_4_)_2_·6H_2_O (25.4 g) were dissolved in 500 mL of bi-distilled water. Solution (B) was prepared by dissolving 50 g of NaOH in 500 mL of water. Solution (A) was gradually added dropwise to solution (B) while vigorously stirring. After being stirred continuously at 60 °C for 2 h, the mixture was cooled to room temperature naturally. The black precipitate that had formed during the reaction was collected and washed several times with bi-distilled water and ethanol. The precipitate was then dried overnight in a 60 °C oven.

A nanocomposite of Fe_3_O_4_ and VCO was synthesized using the procedure detailed by Mohamad et al. (2019). In this method, 27.6-g Fe_3_O_4_ NPs were added to 30-mL pure oil CO while stirring for 30 min. An ultrasonic generator (Sonics Vibracell with probe, SONICA-2200 E, 20 kHz frequency, and a power of 750 W, USA) was used to ensure well dispersion of Fe_3_O_4_ in the oil sample and to reduce the agglomeration. The sonication was carried out for 1 h. In total, 30 min of resting time at ambient temperature was given to the oil samples before it was subjected to drying process in an oven for 48 h at 85 °C.

### Synthesis of chitosan and glutathione-chitosan nanocomposite

Chitosan NPs were synthesized using the ionotropic gelation method, following a procedure similar to the one described by Ayodele et al. (2022) with minor adjustments. Initially, a precise amount of 4.1 g of pure CS was weighed and dissolved in 600 mL of a 2% acetic acid solution. Separately, 10.1 g of sodium tripolyphosphate was weighed and dissolved in 150 mL of distilled water. The sodium tripolyphosphate solution was gradually added drop by drop to the CS solution, and the resulting mixture was left undisturbed for 24 h to fully equilibrate. Subsequently, the mixture underwent filtration, and the remaining residue was washed multiple times with distilled water. Lastly, the residue was dried in an oven at 45 °C, resulting in the formation of CS NPs.

The GSH-CS nanocomposite was prepared using the wet impregnation method (Said et al. 2016, Said and Abd El-Aal 2018). In this approach, a solution containing 0.1 g of GSH dissolved in a small quantity of distilled water was prepared. The GSH solution was then cautiously combined with 0.4 g of CS NPs until a homogeneous paste was formed. Subsequently, the mixture was dried in an oven at 50 °C for 24 h.

### Nanocomposites characterization techniques

X-ray diffraction spectroscopy (XRD) was employed to determine the structure of the nanocomposites. The XRD analysis was conducted using a PW 2103 XRD apparatus manufactured by Philips in the Netherlands, with Cu-Kα radiation (*ʎ* = 0.1542 nm). The scanning range for the samples spanned from 4 to 80°. To investigate the functional groups, present in the nanocomposites, Fourier transform infrared spectroscopy (FTIR) was conducted. The FTIR measurements were performed using a Nicolet spectrophotometer, model 6700. For each analysis, 1 mg of the sample was mixed with 100 mg of KBr (spectral grade) and ground together in an agate mortar. The resulting mixture was then compressed into thin slices and placed into the FTIR spectrometer. The sizes of the NPs were determined using transmission electron microscopy (TEM) with a JEOL Model JSM-5400 LV microscope manufactured by Joel in Tokyo, Japan.

### Evaluation of kidney functions

The renal function markers serum creatinine (Cat. no. 234–000), uric acid (Cat. no. 223–004), and blood urea nitrogen (Cat. no. UR 21–10) were assessed using kits from Schiffgraben in Hannover, Germany, following the specified technique. The parameters listed above can be measured using a spectrophotometer.

### Oxidative stress markers

Spectrophotometric analysis of MDA (Cat. no. MD 25–28) and GSH (Cat. no. GR 25–11) was performed in kidney tissue samples using kits manufactured by Schiffgraben in Hannover, Germany.

### Assessment of interleukin 1 *beta* and tumor necrosis factor alpha concentrations in tissues

In order to test inflammatory markers, kidney tissues were homogenized by centrifuging at 12,000 rpm for 1–2 min on ice. A 10% homogenate was then generated by adding phosphate buffer to the tissues. The supernatant was obtained by centrifuging homogenized tissue samples at 5000 rpm for 30 min at + 4 °C. The biochemical analysis of the groups involved measuring the amounts of IL-1β and TNF-α in the supernatants using ELISA kits specifically designed for rats (Cat. no. E-EL-R0012, Elabscience; Cat. no. E-EL-R2856, Elabscience). All measurements were taken following the guidelines provided by the manufacturer.

### Acute renal failure selective biomarker evaluation

The proximal tubule apical membrane of injured rat kidneys expresses kidney injury molecule-1, a type-1 transmembrane protein that is ordinarily absent. To test for KIM-1, a commercially available ELISA kit was used (Cat. no. E-EL-R3019, Sunlong Biotechnology, Shangyi, China) as stated and in accordance with the manufacturer's instructions (Bonventre 2008).

This particular lipocalin, a member of the neutrophil gelatinase superfamily, is abundantly produced in the injured kidneys of rats, particularly in the proximal convoluted tubule. An ELISA kit that is commercially available was used to test NGAL. (Cat. no. E-EL-R0662, Sunlong Biotechnology, Shangyi, China) (Sabra et al. 2023).

### Histopathological and immunohistochemical assessment

Using 10% neutral buffered formalin, kidney tissue samples were fixed. After that, it is dehydrated using progressively stronger alcohols, cleared with xylene, and finally embedded in paraffin. Hematoxylin and eosin staining of 5-µm-thick tissue sections.

The immunohistochemical staining procedures were done as described by Khalil et al. (2020). The sections were dewaxed and then submerged in an antigen retrieval solution containing a 0.05 M citrate buffer with a pH of 6.8. The next step was to apply protein block and 0.3% H_2_O_2_ to these regions. The next step was to incubate the sections with anti-NF-κB P65 (Santa cruz, Cat# (F-6): sc-8008, 1:100 dilution). After rinsing with phosphate buffered saline, they were incubated with a goat anti-rabbit secondary antibody (Cat# K4003, EnVision + ™ System Horseradish Peroxidase Labelled Polymer; Dako) for 30 min at room temperature. Slides were visualized with DAB kit and eventually stained with Mayer's hematoxylin as a counter stain. The staining labelling indices of the antibodies were presented as a percentage of positive cells per 1000 cells. We took the mean ± SE for each group after counting the number of immunopositive cells in five distinct microscopic regions on each slide (Sabra et al. 2024b).

### Statistical analysis

We checked the data for the normality of distributions for every parameter that was examined (Shapiro–Wilk test, *p* > 0.05). Statistical significance was assessed by one-way ANOVA for independent groups, followed by Bonferroni’s multiple comparison test. *P* ≤ 0.05 was considered statistically significant. Data are presented as means ± SE. Graph Pad prism® software (version 8) was used in order to do these statistical analyses.

## Results

### Characterization of Fe_3_O_4_-VCO nanocomposite

**Fig. 1:**
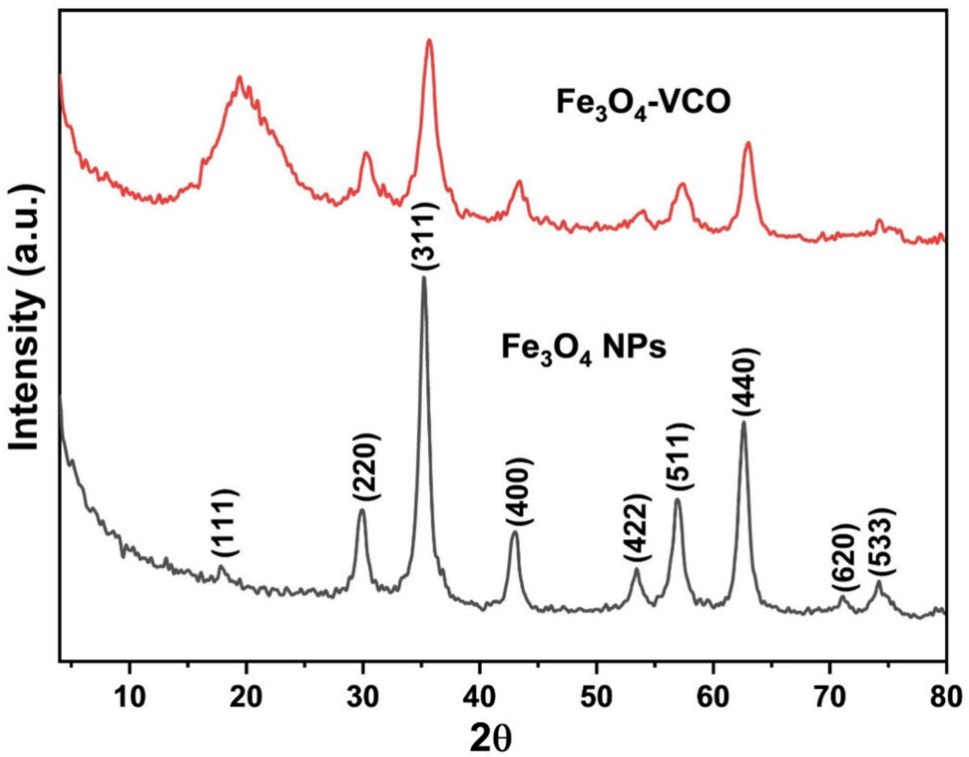
XRD patterns of iron oxide (Fe_3_O_4_) NPs and Fe_3_O_4_-virgin coconut oil (VCO) nanocomposite

**Fig. 2:**
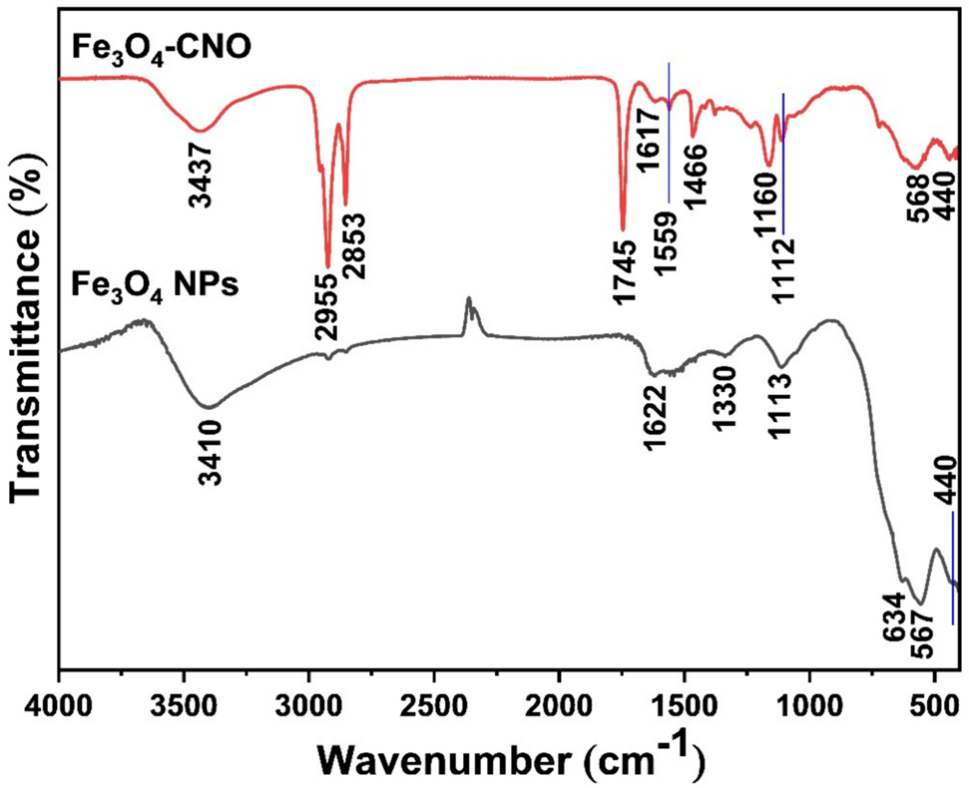
FT-IR spectra of iron oxide (Fe_3_O_4_) NPs and Fe_3_O_4_-virgin coconut oil (VCO) nanocomposite

**Fig. 3:**
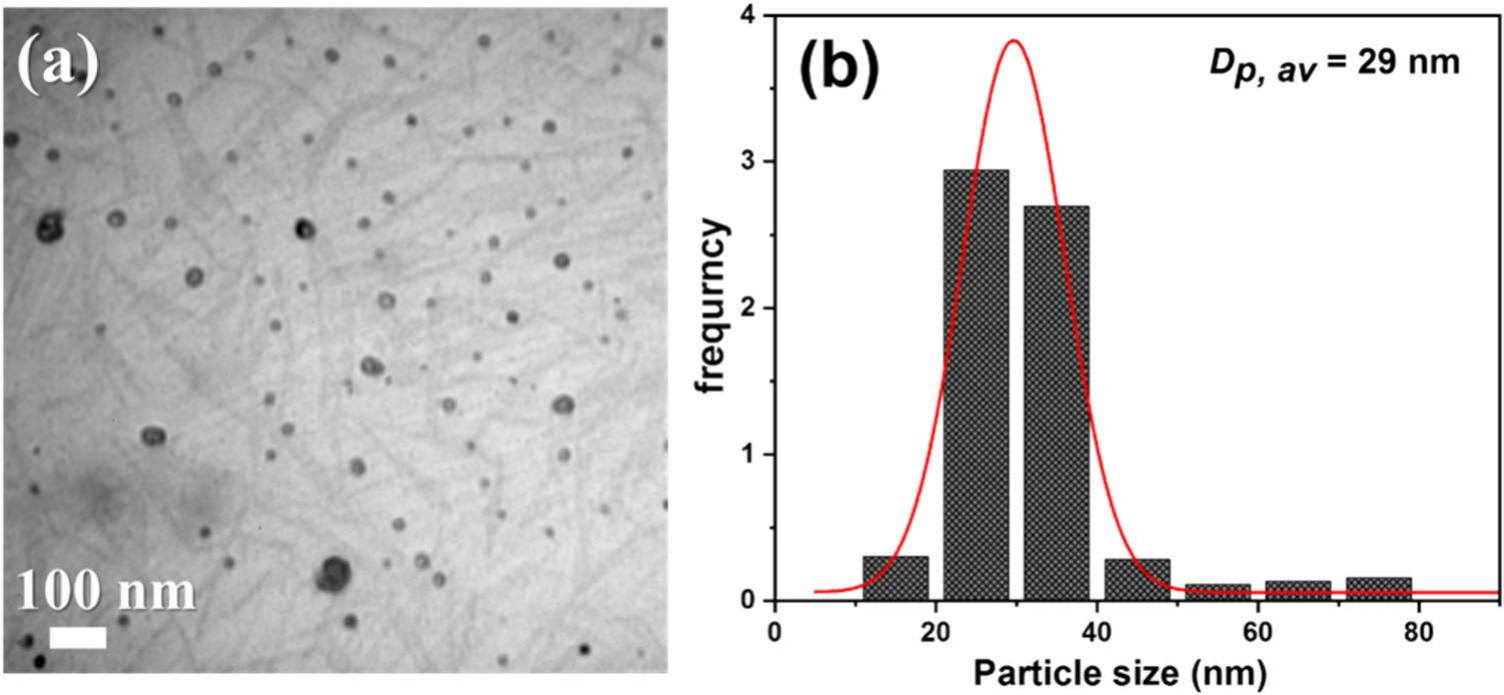
**a** TEM image and **b** particle size distribution of iron oxide-virgin coconut oil nanocomposite

The XRD profiles of both pure Fe_3_O_4_-NPs and Fe_3_O_4_-VCO nanocomposite are shown in Fig. 1. While Fig. 2 illustrates the FT-IR spectra of both pure Fe_3_O_4_ NPs and the Fe_3_O_4_-VCO nanocomposite. The TEM micrograph and the particle size distribution of Fe_3_O_4_-VCO nanocomposite are displayed in Fig. 3.

### Characterization of GSH-CS nanocomposite

**Fig. 4:**
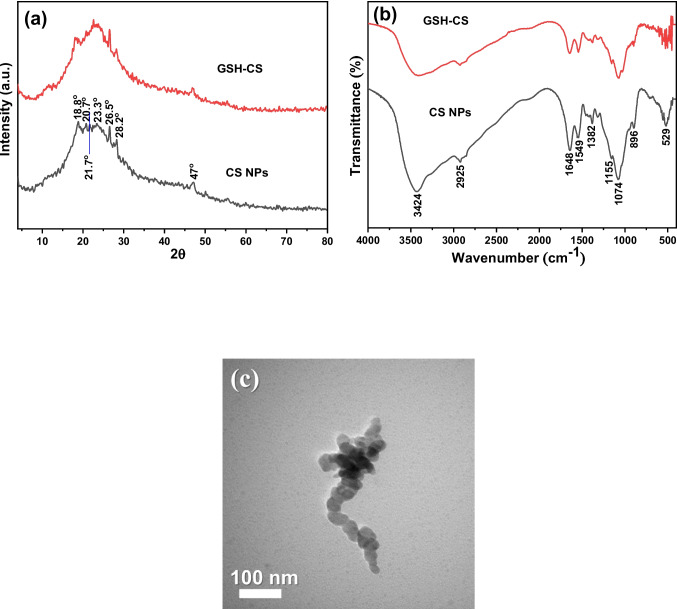
**a** XRD patterns, **b** FTIR spectra of CS NPs and glutathione (GSH)-chitosan (CS) nanoparticles (NPs), and **c** TEM image of GSH-CS NPs

The XRD patterns and the FTIR spectra of CS NPs and the GSH-CS nanocomposite are depicted in Fig. 4a, b, while Fig. 4c shows the TEM image of GSH-CS nanocomposite.

### TEM micrograph and the particle size distribution of Fe_3_O_4_-CO nanocomposite

In Fig. 3a, it can be clearly seen that the Fe_3_O_4_ NPs (black spherical particles) were well dispersed on the surface of fibrous-like particles (VCO). The histogram analysis (Fig. 3b) indicated that the average particle size of Fe3O4 NPs was 29 nm with the narrowest size distribution.

### XRD, FTIR, and TEM micrograph of glutathione-chitosan nanocomposite

The XRD pattern of CS NPs exhibited distinct reflections at 2θ values of 18.8°, 20.7°, 21.7°, 23.3°, 26.5°, 28.2°, and 47°. These reflections indicate the amorphous nature of the CS NPs, which can be attributed to the crosslinking mechanism between the reactive functional groups of CS and TPP (Joseph et al. 2016). In the XRD pattern of the GSH-CS nanocomposite, some characteristic peaks of CS showed slight reductions, and a new peak at a 2θ value of 22.6°, attributed to GSH, was observed. This result suggests successful loading of GSH onto the surface of CS NPs. The average crystallite sizes of CS NPs and the GSH-CS nanocomposite were calculated to be 25.4 nm and 17.1 nm, respectively, using the Sherrer equation (Fig. 4a).

The FTIR spectrum of CS NPs displays several characteristic bands (Fig. 4b), including peaks at 3424 cm^−1^ (overlapping of O–H and N–H stretches), 2925 cm^−1^ (asymmetric CH2 stretching vibration from the pyranose ring), 1648 cm^−1^ (C = O asymmetric stretching), 1549 cm^−1^ (NH2 groups), 1382 cm^−1^ (C-H bending), 1321 cm^−1^ (N–H wag of primary and secondary amine), 1155 cm^−1^ (C–O–C linkage), 1074 cm^−1^ (P = O stretching), 896 cm^−1^ (antisymmetric stretching vibration of C–O–C bridges assigned to the glucopyranose ring in the CS matrix), and 529 cm^−1^ (OH out-of-plane bending vibration). These bands provide valuable information about the functional groups present in CS NPs. The FTIR spectrum of the GSH-CS nanocomposite exhibits bands that are very similar to those observed in the case of CS NPs, but with slightly decreased intensities. Additionally, new bands were observed at 452–596 cm^−1^, which are attributed to the C-S stretching vibrations of GSH according to Arocikia Jency et al. (2018). This observation serves as evidence of successful loading of GSH onto the surface of CS NPs.

The TEM image (Fig. 4c) of GSH-CS nanocomposite showed semi-spherical particles with an average size of 38.6 nm.

### The impact of coconut oil, GSH, and their nanoparticle formulations on blood urea, creatinine, and uric acid levels in rats induced with ARF

**Fig. 5:**
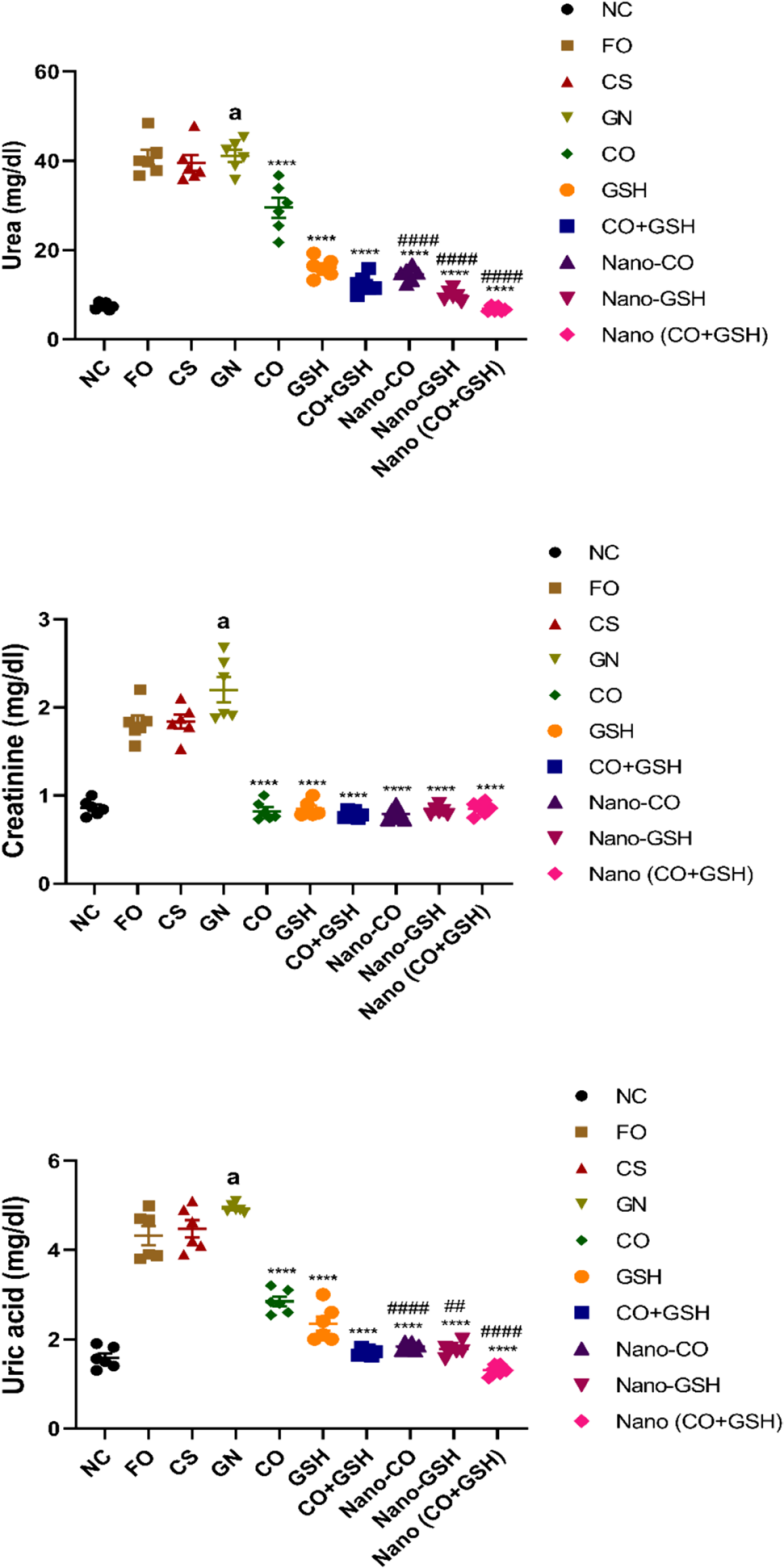
Acute renal failure caused by gentamicin (GN) in rats: effects of conventional, nanoparticle, and combination forms of glutathione (GSH) and coconut oil (CO) on blood levels of urea, creatinine, and uric acid. Data are the means ± SEM (*n* = 6). ^a^*p* < 0.0001 as compared with the negative control group (NC). ^****^p < 0.0001 as compared to the gentamicin-treated group. ^##^*p* < 0.01, and.^####^*p* < 0.0001 when contrasted with the related nanoparticle category. Note: FO (ferric oxide), CS (chitosan), nano-CO (coconut oil nanoparticles), nano-GSH (glutathione nanoparticles), nano-(CO + GSH) (coconut oil-glutathione nanoparticle combinations)

The gentamicin group had significantly elevated serum urea, creatinine, and uric acid levels compared to the negative control group (*p* < 0.0001). Rats treated with CS and Fe_3_O_4_ NPs showed no significant changes in serum urea, creatinine, and uric acid levels compared to rats with ARF (F_2,15_ = 0.2608, F_2,15_ = 4.058, F_2,15_ = 3.578, respectively). In addition, when comparing rats induced with ARF to animals given either conventional or NPs of CO, GSH, or their combination, it was observed that the latter groups had dramatically reduced levels of serum urea (F_6,35_ = 115.2), creatinine (F_6,35_ = 74.07), uric acid levels (F_6,35_ = 221.3), (*p* < 0.0001). The decline was most pronounced in rats treated with NPs form of the CO, GSH, and their combination in comparison with their conventional forms (Fig. 5).

### The impact of coconut oil, GSH, and their nanoparticle formulations on tissue malondialdehyde and reduced GSH levels in rats induced with ARF

**Fig. 6:**
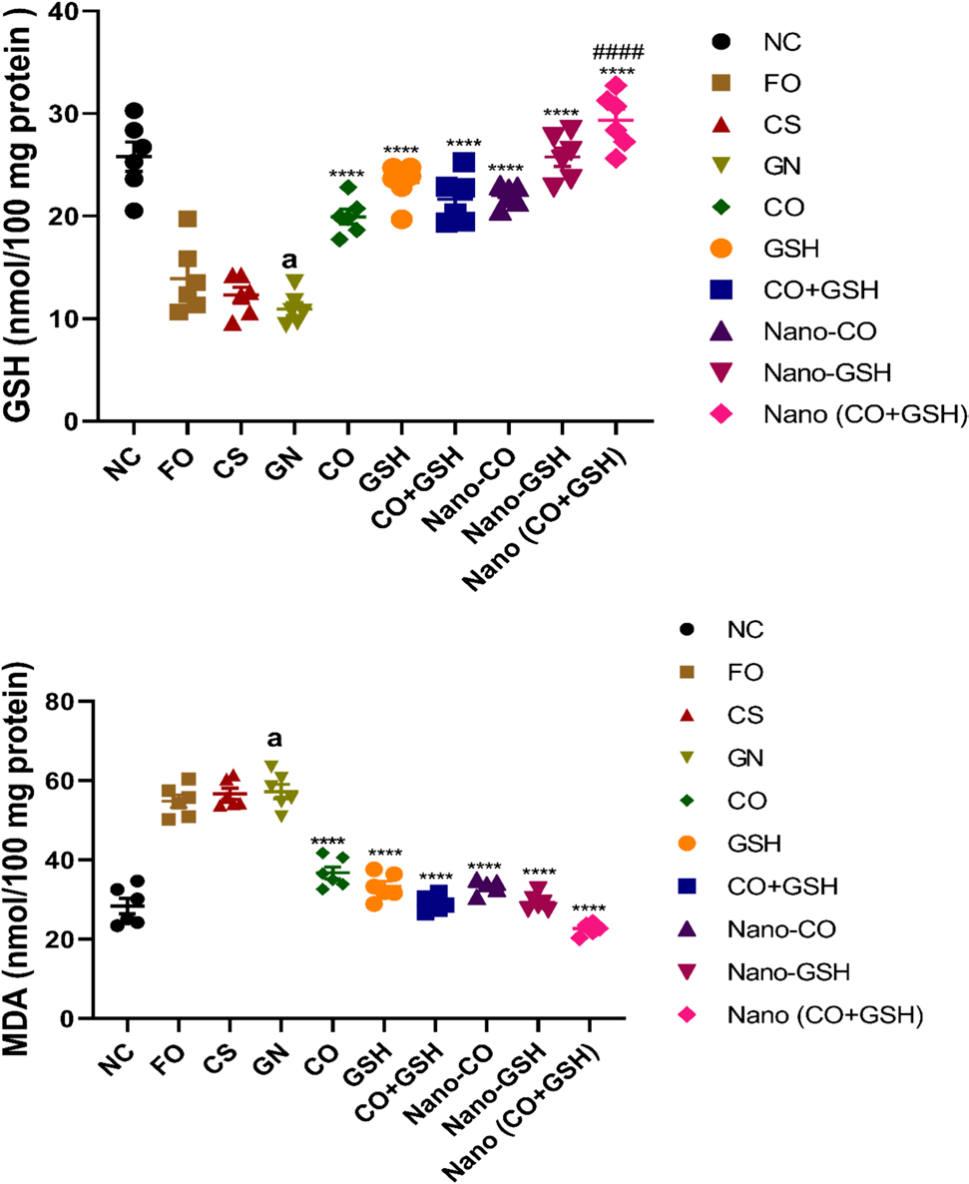
Acute renal failure caused by gentamicin (GN) in rats: effects of conventional, nanoparticle, and combination forms of glutathione and coconut oil (CO) on tissue levels of glutathione (GSH) and malondialdehyde (MDA). Data are the means ± SEM (*n* = 6). ^a^*p* < 0.0001 as compared with the negative control group (NC). ^****^p < 0.0001 as compared to the gentamicin-treated group.^#^*p* < 0.05, ^##^*p* < 0.01, and.^###^*p* < 0.001 when contrasted with the related nanoparticle category. Note: FO (ferric oxide), CS (chitosan), nano-CO (coconut oil nanoparticles), nano-GSH (glutathione nanoparticles), nano-(CO + GSH) (coconut oil-glutathione nanoparticle combinations)

Rats administered with gentamicin demonstrated a significant increase in tissue MDA levels, (*p* < 0.0001) and a substantial decrease in GSH levels, (*p* < 0.0001) compared to the negative control group, as illustrated in Fig. 6. Rats treated with CS and Fe_3_O_4_ did not show significant differences in MDA (F_2,15_ = 0.5944) and GSH (F_2,15_ = 2.308) levels compared to the gentamicin group. When traditional treatments or NP versions of CO, GSH, or their combination were used, there was a dramatic decrease in tissue MDA levels (F_6,35_ = 89.48), (*p* < 0.0001), while GSH (F_6,35_ = 49.73) levels significantly increased (*p* < 0.0001) compared to ARF positive control group. In comparison to animals that received the same conventional treatments, CO-GSH NPs combination exhibited a significant increase in tissue GSH (*p* < 0.0001) and a notable decrease in tissue MDA levels.

### The impact of coconut oil, GSH, and their nanoparticle formulations on pro-inflammatory cytokines (TNF-α and IL-1β) levels in rats induced with ARF

When juxtaposed with the negative control group, the rats that received gentamicin showed a significant increase in the levels of tissue TNF-α, (*p* < 0.0001) and IL-1β, (*p* < 0.0001). However, when these results were contrasted with the ARF-positive control group, no noticeable changes were observed in the levels of tissue TNF-α (F_2,15_ = 1.507) and IL-1β (F_2,15_ = 0.2378) in the rats treated with CS or Fe_3_O_4_.

**Fig. 7:**
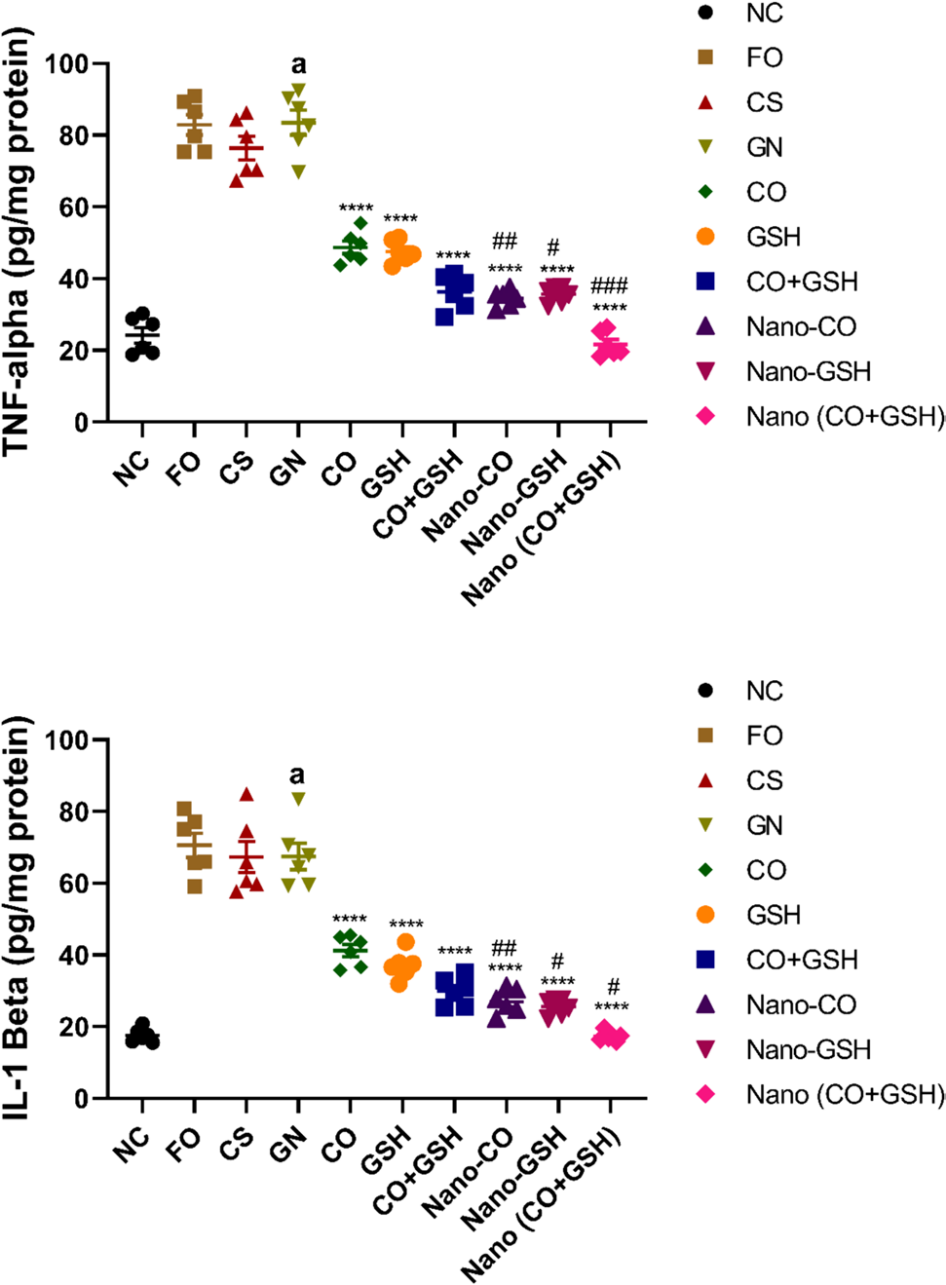
Acute renal failure caused by gentamicin (GN) in rats: effects of conventional, nanoparticle, and combination forms of glutathione (GSH) and coconut oil (CO) on tissue levels of interleukin 1 beta (IL-1β) and tumor necrosis factor alpha (TNF-α). Data are the means ± SEM (*n* = 6). ^a^*p* < 0.0001 as compared with the negative control group (NC). ^****^p < 0.0001 as compared to the gentamicin-treated group.^##^*p* < 0.01, and.^###^*p* < 0.001 when contrasted with the related nanoparticle category. Note: FO (ferric oxide), CS (chitosan), nano-CO (coconut oil nanoparticles), nano-GSH (glutathione nanoparticles), nano-(CO + GSH) (coconut oil-glutathione nanoparticle combinations)

In opposition to the ARF-induced group, a significant reduction (*p* < 0.0001) in the levels of tissue TNF-α (F_6,35_ = 113.6) and IL-1β (F_6,35_ = 75.70) was observed when either the conventional or NP versions of CO, GSH, or their combination were administered. It is of particular interest to note that, when compared to rats that received their respective conventional drugs, the animals treated with nanoparticle versions of CO or GSH, or their combination, displayed a significant reduction in the levels of tissue TNF-α and IL-1β (*p* < 0.01), as illustrated in Fig. 7.

### The impact of coconut oil, GSH, and their nanoparticle formulations on tissue kidney injury molecule-1 and neutrophil gelatinase–associated lipocalin levels in rats induced with ARF

Compared to the negative control group, rats given gentamicin displayed significantly elevated levels of tissue KIM-1 (*p* < 0.0001) and NGAL (*p* < 0.0001). However, in comparison with the ARF-positive control group, rats that were treated with CS or Fe_3_O_4_ did not show discernible changes in KIM-1 (F_2,15_ = 2.985) and NGAL (F_2,15_ = 1.415) tissue levels.

**Fig. 8:**
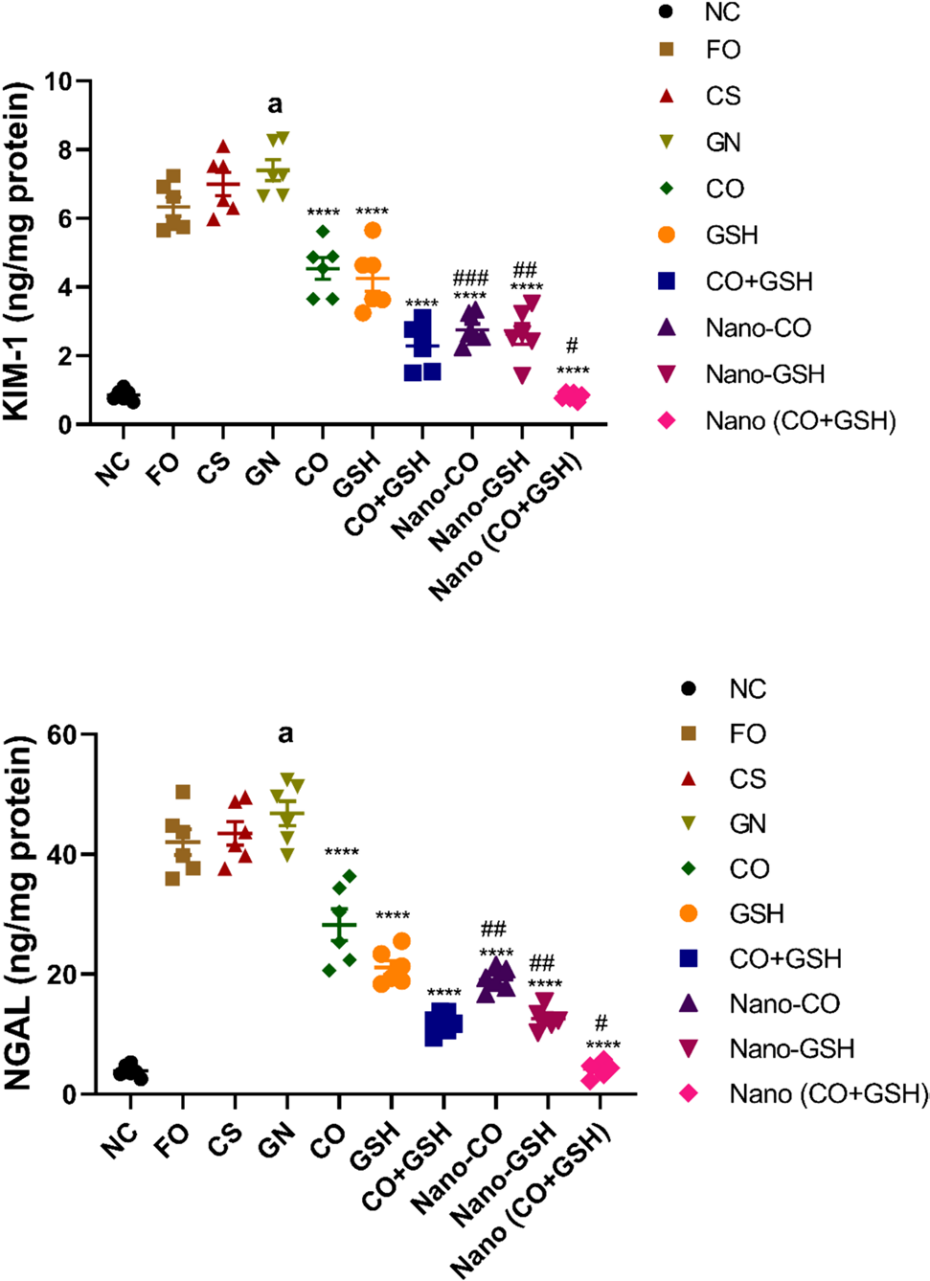
Acute renal failure caused by gentamicin (GN) in rats: effects of conventional, nanoparticle, and combination forms of glutathione (GSH) and coconut oil (CO) on tissue levels of kidney injury molecule-1 (KIM-1) and neutrophil gelatinase-associated lipocalin (NGAL). Data are the means ± SEM (*n* = 6). ^a^*p* < 0.0001 and ^b^*p* < 0.001 as compared with the negative control group (NC). ^****^p < 0.0001 as compared to the gentamicin-treated group.^##^*p* < 0.01, ^###^*p* < 0.001, and.^###^*p* < 0.001 when contrasted with the related nanoparticle category. Note: FO (ferric oxide), CS (chitosan), nano-CO (coconut oil nanoparticles), nano-GSH (glutathione nanoparticles), nano-(CO + GSH) (coconut oil-glutathione nanoparticle combinations)

In contrast to the ARF-induced group, both the conventional and NP versions of CO, GSH, or their combination resulted in a substantial reduction (*p* < 0.0001) in tissue KIM-1 (F_6,35_ = 59.87) and NGAL (F_6,35_ = 39.99) levels. It is noteworthy that, compared to rats treated with their respective conventional drugs, animals that were administered with nanoparticle versions of CO or GSH, or their combination, exhibited a notable reduction in tissue KIM-1 and NGAL levels, as depicted in Fig. 8.

### Alterations in histopathology observed in acute renal failure caused by gentamicin

**Fig. 9:**
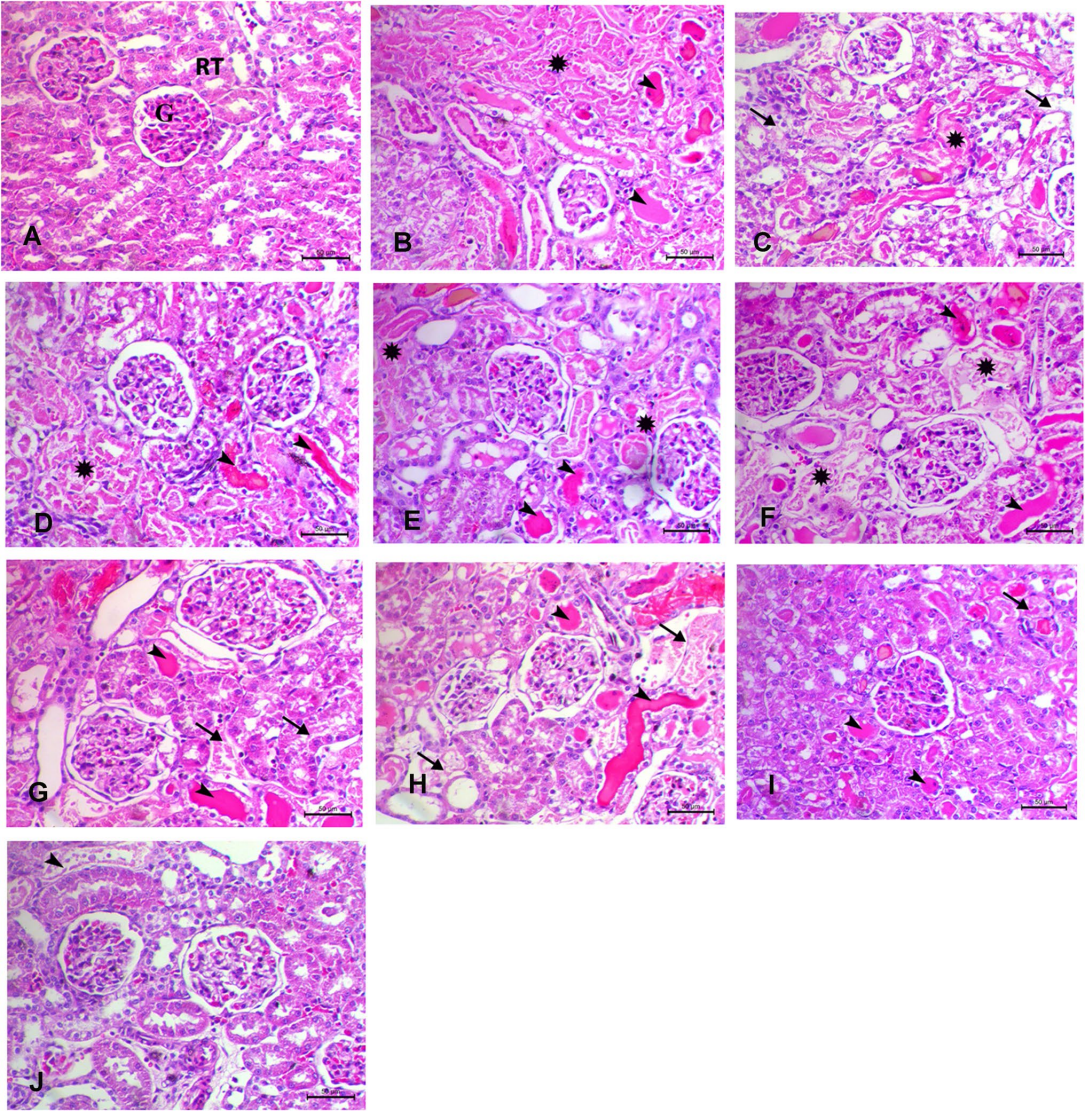
H&E staining in rat kidney of different treated groups. Scale bar = 50 μm. **A** Control group showing normal renal histo-architecture of normal renal glomerulus (G) and tubules (RT). **B** Gentamicin group showing massive coagulative tubular necrosis with loss of arichitecture of renal tubules (asterick) associated with homogeneous esinophilic proteins-filled fluid within the lumen of the renal tubules (arrowheads). **C** and **D** Gentamicin + chitosan and gentamicin + Fe_3_O_4_ groups showing multifocal tubular necrosis (arrow) with some hydropic degeneration(asterick) associated with proteins-filled fluid within the lumen of the renal tubules (arrowheads). **E** and **F** Gentamicin + glutathione and gentamicin + VCO groups showing some improvement of tubular architecture with a few focal of tubular necrosis (astericks) and protein-filled fluid within the lumen of the renal tubules (arrowheads). **G** and **H** Gentamicin + nano-glutathione and gentamicin + nano-VCO groups showing significant improvement of tubular architecture with a few degeneration of the tubular epithelium (arrows) and presence of protein-filled fluid within the lumen of some renal tubules (arrowheads). **I** and **J** Gentamicin + combination and gentamicin + nano-combination groups marked improvement of tubular architecture with minute degenerative changes (arrow) with marked decrease of protein-filled fluid within the lumen of renal tubules (arrowheads)

As shown in Table 1, the histopathological results for each group were summarized according to the lesion score. Kidneys in the negative control group showed normal architecture upon examination. Tubular and glomerular alterations were seen in the gentamicin-induced ARF group. There were noticeable glomerular alterations and tubular abnormalities, including homogeneous eosinophilic protein–filled fluid inside the renal tubule lumen and significant coagulative necrosis. In addition to the identical gentamicin-induced ARF lesion and significant hydropic vacuolation of the renal tubular epithelial lining, the groups treated with CS and Fe3O4 exhibited similar results. Although the renal tubule architecture was partially restored and there was reduced protein fluid buildup within the lumen, traditional and NPs forms of GSH and VCO showed less tubular improvement. There was a significant improvement in the tubular cells and a full recovery of the renal tubule architecture in the group that used standard GSH-VCO combination solely, as shown in Fig. 9. Solely, a little amount of protein fluids accumulated within the tubule lumen was seen in this group. In addition, NPs forms of GSH-VCO combination showed marked improvement of the tubular cells with complete recovery of the architecture of renal tubules.

**Table 1:** Summarized the lesion score of the examined groups

Lesion group	Coagulative necrosis of renal tubules	Vacuolar degeneration of renal tubules	Loss of tubule architecture	Protein fluid within the tubular lumen
Control-ve	-	-	-	-
Gentamicin-induced ARF	+ + +	+ + +	+ + +	+ + +
Gentamicin+chitosan	+ +	+ +	+ +	+ + +
Gentamicin+iron oxide	+ +	+ +	+ +	+ + +
Gentamicin+glutathione	+	+	+	+ +
Gentamicin + VCO	+	+	+	+ +
Gentamicin + nano-glutathione	-	+	+	+ +
Gentamicin + nano-VCO	-	+	+	+ +
Gentamicin + combination	-	+	-	+
Gentamicin + nano combination	-	-	-	+

^−^No lesions, ^+^lesions present in 2–3 sections, ^++^lesions present in 4–7 sections, ^+++^lesions present in 8–10 sections. *VCO* Virgin coconut oil

### Immunohistochemistry changes in nuclear factor-kappa B expression in acute renal failure

**Fig. 10:**
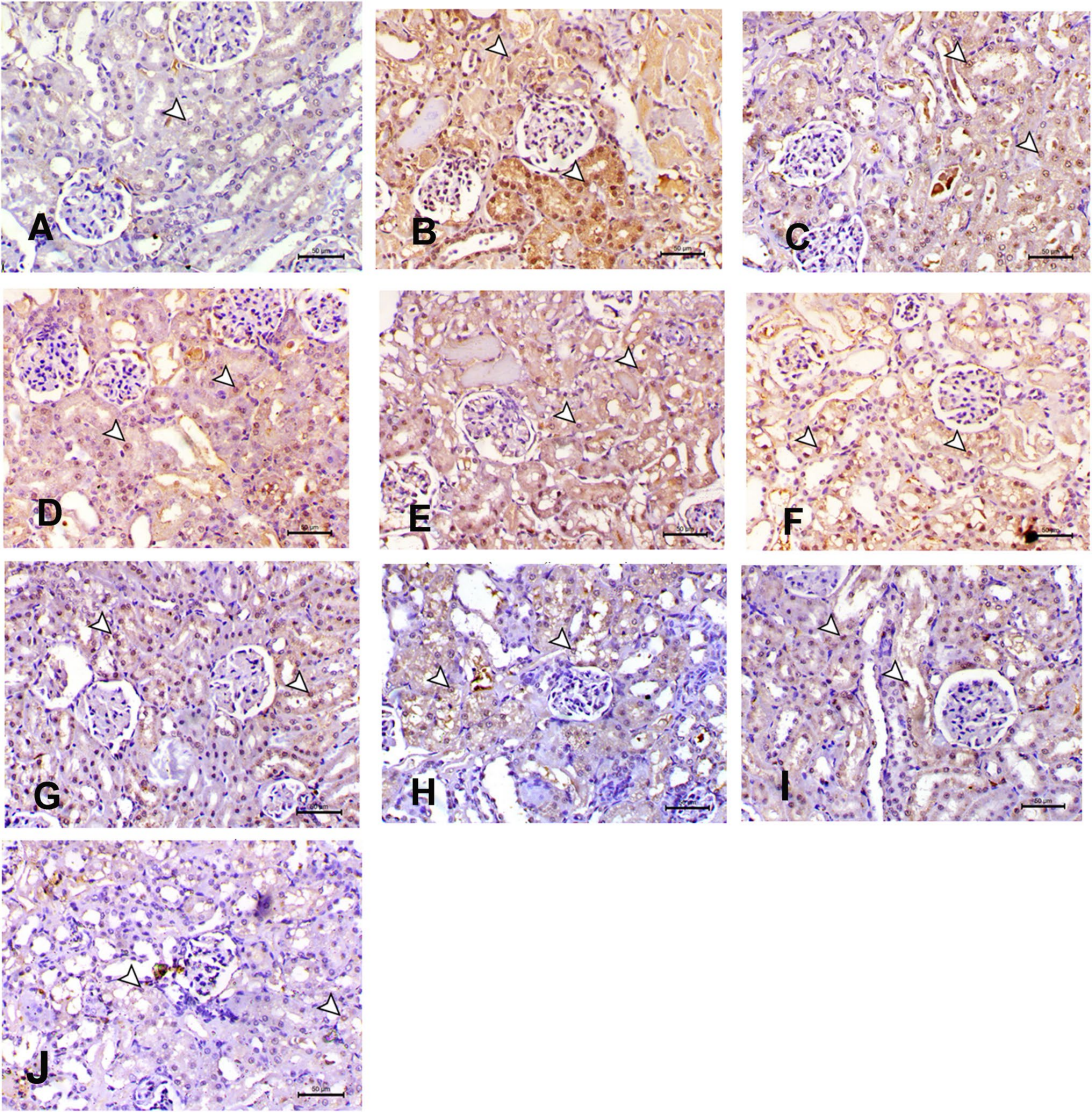
Immunohistochemical staining of nuclear factor-kappa B (NF-κB) in the kidneys of rats was stained using immunohistochemistry. There was a notable rise in NF-κB immunoreactivity in the group that was treated with gentamicin. The groups that received either the conventional or nanoform of glutathione-virgin coconut oil (VCO)showed very little expression. In the negative control group, no expression was detected in rat kidney. **A** Control negative group showing no expression of NF-κB. **B** Gentamicin-induced ARF group showing a significant increase in NF-kB immunoreactivity in the cytoplasm of renal tubular epithelial cells. Brown color indicates NF-κB positivity. **C** Gentamicin-chitosan and **D** gentamicin-Fe_3_O_4_ groups showing less increase in NF-κB immunostaining. **E** Gentamicin + glutathione and **F** Gentamicin + VCO groups showing non-significant reduction in NF-κB immunostaining. **G** Gentamicin + nano-glutathione and **H** gentamicin + nano-VCO groups showing noticeable decrease in NF-κB immunostaining. **I** Gentamicin-combination and **J** gentamicin-nano-combination groups showing rare expression of NF-κB immunostaining

The NF-κB in the kidneys of rats was stained using immunohistochemistry. There was a notable rise in NF-κB immunoreactivity in the group that was treated with gentamicin. The groups that received either the conventional or nanoform of GSH-VCO showed very little expression. In the negative control group, no expression was detected (Fig. 10 and Table 2).

**Table 2:** Effects of gentamicin-induced ARF, glutathione, virgin coconut oil in normal and nanoforms treatment on the percentage expression of nuclear factor-kappa B (NF-κB) in the kidney of rats

Groups	Control-ve	Gentamicin-induced ARF	Gentamicin-chitosan	Gentamicin-Fe_3_O_4_	Gentamicin-glutathione	Gentamicin-VCO	Gentamicin-nano glutathione	Gentamicin-nano-VCO	Gentamicin + combination	Gentamicin + nano-combination
NF-κB	N^a^	49.29±3.1^b^	28.45±2.4^b^	30.7±3.3^b^	14.43±2.2^c^	11.30±1.6^c^	12.19±0.9^c^	11.26±1.1^c^	12.73±1.2^c^	4.52±0.4^d^

^a^^,^^b,c,d^Means ± SE values are not sharing a common superscript letter differ significantly at *P* < 0.05. *VCO* virgin coconut oil

## Discussion

Acute kidney injury (AKI) is a condition characterized by a rapid decline in glomerular filtration rate (GFR), leading to impaired renal function. Despite advancements in medical management, AKI remains associated with significant morbidity and mortality, with etiologies varying based on socioeconomic context (Bellomo et al. 2012).

The NPs employed in this study demonstrate physicochemical characteristics—such as size, morphology, and charge density—that align with previous research findings (Hamdy et al. 2022). Their small dimensions and spherical shape enhance their capacity for effective drug delivery and precise targeting in therapeutic applications.

The XRD pattern of pure Fe_3_O_4_ displayed distinct peaks at 2θ values of 17.8°, 29.8°, 35.2°, 43°, 53.4°, 56.9°, 62.6°, 71.1°, and 74.2°. These peaks correspond to the (111), (220), (311), (400), (422), (511), (440), (620), and (533) crystallographic planes of the inverse spinel Fe_3_O_4_ structure (JCPDS No. 65–3107) (He et al. 2013). The absence of peaks from any other impurities confirms the high purity of this sample, while the XRD pattern of the Fe_3_O_4_-VCO nanocomposite exhibited reflections at the same 2θ values as that observed in the pattern of pure Fe_3_O_4_ NPs, with lower intensities. In addition, a new peak was observed at 2θ value of 19.4°, which may be attributed to the CO. This result confirmed that the successful synthesis of Fe_3_O_4_-VCO nanocomposite (Liu et al., 2023). The average crystallite sizes of Fe_3_O_4_ NPs and the nanocomposite were evaluated using Scherrer equation using all the detected peaks (Alamri et al. 2022; Ali et al. 2022; Mousa et al. 2024) and were found to be equal to 17.5 and 13.7 nm, respectively.

In the case of pure Fe_3_O_4_ NPs, the bands observed at 3410 and 1622 cm^−1^ correspond to the stretching and bending vibrations of O–H, respectively (Najjar et al. 2022). The two bands that detected at 1330 and 1113 cm^−1^ are attributed to deionized water that is used as a solvent (Chaki et al. 2015). The presence of two bands at 634 and 567 cm^−1^ provides evidence of the formation of magnetic NPs (Pham et al. 2016). Furthermore, the band at 567 cm^−1^ can be attributed to the Fe–O stretching vibration of the tetrahedral sites in the spinel structure, while the band at 441 cm^−1^ is associated with the tetrahedral and octahedral sites (He et al. 2013). In the case of the Fe_3_O_4_-VCO nanocomposite, various bands were observed, the bands at 568 and 440 cm^−1^ are related to the presence of Fe_3_O_4_ in the nanocomposite. The characteristics bands of VCO can be observed at 2955–2853 cm^−1^ (= C-H (alkene) and C-H (alkane) stretching vibrations (Ong et al. 2021)), 1745 cm^−1^ (C = O stretching of ester (Brandão et al. 2021)), and 1617 cm^−1^ (C = C stretching mode). In addition, the bands at 1559 cm^−1^ (CH_2_ bending vibration (Yunus et al. 2009)), 1466 cm^−1^ (CH_3_ bending vibration (Yunus et al. 2009)), 1160 cm^−1^ (C–O–C bond stretching vibration (Saraç et al. 2019)), 1234, 1112 cm^−1^ (C-O stretching mode (Md Saari et al. 2020)), and 721 cm^−1^ (CH_2_ asymmetric deformation (Brandão et al. 2021)). This result also confirms that the nanocomposite was successfully prepared (Fig. 2).

In Fig. 3a, it can be clearly seen that the Fe_3_O_4_ NPs (black spherical particles) were well dispersed on the surface of fibrous-like particles (VCO). The histogram analysis (Fig. 3b) indicated that the average particle size of Fe_3_O_4_ NPs obtained by counting 100 particles from different images was 29 nm with the narrowest size distribution.

The XRD pattern of CS NPs exhibited distinct reflections at 2θ values of 18.8°, 20.7°, 21.7°, 23.3°, 26.5°, 28.2°, and 47°. These reflections indicate the amorphous nature of the CS NPs, which can be attributed to the crosslinking mechanism between the reactive functional groups of CS and TPP (Joseph et al. 2016). In the XRD pattern of the GSH-CS nanocomposite, some characteristic peaks of CS showed slight reductions, and a new peak at a 2θ value of 22.6°, attributed to GSH, was observed. This result suggests successful loading of GSH onto the surface of CS NPs. The average crystallite sizes of CS NPs and the GSH-CS nanocomposite were calculated using all the detected peaks to be 25.4 nm and 17.1 nm, respectively, using the Sherrer equation (Fig. 4a).

The FTIR spectrum of CS NPs displays several characteristic bands (Fig. 4b), including peaks at 3424 cm^−1^ (overlapping of O–H and N–H stretches), 2925 cm^−1^ (asymmetric CH_2_ stretching vibration from the pyranose ring), 1648 cm^−1^ (C = O asymmetric stretching), 1549 cm^−1^ (NH_2_ groups), 1382 cm^−1^ (C-H bending), 1321 cm^−1^ (N–H wag of primary and secondary amine), 1155 cm^−1^ (C–O–C linkage), 1074 cm^−1^ (P = O stretching), 896 cm^−1^ (antisymmetric stretching vibration of C–O–C bridges assigned to the glucopyranose ring in the CS matrix), and 529 cm^−1^ (OH out-of-plane bending vibration). These bands provide valuable information about the functional groups present in CS NPs. The FTIR spectrum of the GSH-CS nanocomposite exhibits bands that are very similar to those observed in the case of CS NPs, but with slightly decreased intensities. Additionally, new bands were observed at 452–596 cm^−1^, which are attributed to the C-S stretching vibrations of GSH according to Arocikia Jency et al. (2018). This observation serves as evidence of successful loading of GSH onto the surface of CS NPs.

The TEM image (Fig. 4c) of GSH-CS nanocomposite showed semi-spherical particles with an average size of 38.6 nm obtained by counting 100 particles from different images.

In our investigation, the administration of the drugs under examination significantly reduced both urea, uric acid, and serum creatinine levels in the context of gentamicin-induced AKI. These findings emphasize the reliability of these metrics as indicators of therapeutic improvement in AKI. Notably, the nanoparticulate formulations of VCO, GSH, and their combined regimen demonstrated a substantial reduction in renal injury markers compared to their conventional drug-treated counterparts. The findings from Famurewa et al. (2020) align with our observations. In their study, VCO administration significantly reduced serum creatinine, urea, and uric acid levels in a rat model of gentamicin-induced nephrotoxicity. Similarly, Zunino et al. (1983) reported that GSH administration effectively lowered serum creatinine and urea concentrations in rats exposed to cis-dichlorodiammine platinum (II). These collective results emphasize the therapeutic potential of these agents, particularly in their nanoparticulate formulations, for mitigating renal injury markers in AKI scenarios.

Reactive oxygen species play a crucial role in the pathogenesis of AKI. These oxygen-derived free radicals can initiate lipid peroxidation within cellular membranes, ultimately leading to renal tubular necrosis (Singh et al. 2022). In our investigation, lipid peroxidation emerged as a significant factor contributing to renal damage with a reduction in GSH levels. Notably, the therapeutic regimens involving CO, GSH, and their combined application both in traditional and NP formulations effectively mitigated these detrimental markers. Remarkably, the nanoparticulate versions exhibited enhanced efficacy, highlighting the potential therapeutic utility of our agents in managing AKI.

Our findings align with the research conducted by Famurewa et al. (2020). In their study, the administration of VCO (at a concentration of 10% w/w) significantly reduced MDA levels and increased GSH concentrations in a gentamicin-induced renal disease model. Similarly, Famurewa et al. (2017) reported that VCO administration (at 15% w/w) lowered MDA levels while simultaneously augmenting GSH concentrations in male rats exposed to methotrexate-induced nephrotoxicity. Additionally, Xu et al. (2012) demonstrated that GSH administration following cisplatin exposure resulted in a decline in MDA levels. These collective findings underscore the potential therapeutic benefits of these agents in managing renal injury markers.

Neutrophil gelatinase–associated lipocalin is expressed in various organs. Under normal physiological conditions, its expression in the kidneys, trachea, and gastrointestinal tract remains relatively subdued. However, following ischemic events, NGAL secretion within the thick ascending limb of the renal tubules escalates rapidly (Zhang et al. 2016). Simultaneously, KIM-1 has emerged as a potential biomarker for renal injury, offering utility in detecting and monitoring nephrotoxic agents (Tanase et al. 2019). Although KIM-1 is undetectable in healthy kidneys, its expression significantly increases in the context of renal dysfunction (Ichimura et al. 2004). Notably, studies have highlighted that post-renal injury, KIM-1 mRNA levels exhibit a more pronounced elevation than any other gene (Wu et al. 2017).

In our study, findings demonstrated that interventions involving CO, GSH, and their combined regimen both in traditional and NP formulations resulted in significant reductions in NGAL and KIM-1 concentrations in the gentamicin-induced AKI model. The observed improvements in AKI by our therapeutic agents likely stem from the observed reductions in NGAL and KIM-1 levels. Supporting our results, Matsubara et al. (2019) also reported elevated urinary KIM-1 levels in GSH-depleted mice. Notably, there are no existing studies discussing the effect of CO on KIM-1 and NGAL levels in any AKI-induced model.

Acute kidney injury is triggered by the production of harmful free radicals and inflammatory processes, with elevated levels of inflammatory cytokines like TNF-α, IL-6, and IL-1β (Simmons et al. 2004). Moreover, treatment with gentamicin can lead to a substantial increase in the plasma levels of certain inflammatory cytokines, including TNF-α and IL-1β (Babaeenezhad et al. 2024). Oxidative stress, linked to ARF pathogenesis, can cause mitochondrial dysfunction-related apoptosis, exacerbating renal impairment (Zhan et al. 2013).

In cases of gentamicin-induced ARF, our results were in agreement with those of Babaeenezhad et al. (2024) who found a notable rise in the protein expression of both IL-1β in the renal cortex. Our results could provide an explanation for the amelioration of gentamicin-induced ARF through the administration of CO, GSH, and their combination in both conventional and NP forms, which appears to reduce the protein expression of both IL-1β and TNF-α. The NP forms of CO and GSH resulted in a more pronounced improvement. In line with our observations, Famurewa et al. (2019) (12) showed that the administration of VCO significantly reduced IL-1β, TNF-α, and caspase-3 levels in a rat model of gentamicin-induced nephrotoxicity. Similarly, Junita et al. (2021) reported a decrease in TNF-α levels following the injection of GSH in a male rat peritonitis model.

The current findings also showed that the pathophysiological ameliorating effect of VCO, GSH, and their combination in both conventional and NP forms were accompanied with the inhibition of NF-κB that controls cytokine production and cell survival. Studies have demonstrated that CO may modulate the production of inflammatory cytokines and mediators under inflammatory conditions. Đurašević et al. (2020) reported that VCO decreased the nuclear content of NF-kB in alloxan-induced diabetic rats. Similarly, Asuku (2022) reported that 5% VCO as supplemented diet on sodium benzoate (SB)–induced neurotoxicity in male Wistar rats significantly decreased NF-κB gene expression levels. There are no studies that demonstrated the effect of CO or GSH on NF-kB levels in AKI model.

The histopathological examination of renal tissues confirmed our findings, revealing distinct features such as pronounced tubular necrosis, dilation, cast formation, and evidence of necrotic nuclei. Additionally, within the tubular epithelial cells of the gentamicin-induced AKI cohorts, there was a significant upregulation of NF-ĸB immunoexpression. These observations closely align with the research conducted by (Mahmoud et al. 2021, Mahmoud 2017), both of whom documented elevated levels of NF-ĸB immunoexpression in gentamicin-exposed mice and rats, respectively.

Remarkably, the carrier NPs-CS and -Fe_3_O_4_ did not cause any substantial alterations in renal parameters when compared to the gentamicin group. This suggests that they neither negatively impacted the baseline renal function nor exacerbated injury mechanisms. These findings underscore their potential safety for drug delivery systems (Ma et al. 2020; Merlin and Li 2022).

In summary, the biochemical, histopathological, and immunohistochemical findings from this study robustly endorse the renal protective effects of VCO and GSH, in both their conventional and nanoparticle forms, against gentamicin-induced AKI. The combination of VCO and GSH in their conventional forms demonstrated superior renal enhancement compared to the individual drugs. The nanoparticle forms of these drugs showed superior improvement when used individually or in combination compared to their conventional counterparts. However, it is important to note that there are currently no clinically approved NPs that are specifically designed to target the kidney for therapeutic or imaging purposes. Therefore, additional research is warranted to explore the potential application of nanoparticle-loaded drugs for the treatment of kidney diseases.

## Conclusion

The study shows that administering VCO and GSH, either alone or together, in conventional and nanoparticulate forms, improves the treatment of ARF in a rat model produced by gentamicin. The combined nanoparticulate compositions show significant effectiveness. This observation highlights the possible treatment consequences for managing ARF.

## Supplementary Information

Below is the link to the electronic supplementary material.

Supplementary file1 (PZFX 804 KB)

## Data Availability

All data supporting the findings of this study are available within the paper and its Supplementary Information.

## Abbreviation

AKI: Acute kidney injury
ARF: Acute renal failure
CS: Chitosan
CO: Coconut oil
FTIR: Fourier transform infrared spectroscopy
GSH: Glutathione
IL-1β: Interleukine-1beta
Fe_3_O_4_: Iron oxide
KIM-1: Kidney injury molecule-1
MDA: Malondialdehyde
NPs: Nanoparticles
NGAL: Neutrophil gelatinase–associated lipocalin
NF-κB: Nuclear factor-kappa B
ROS: Reactive oxygen species
GSH: Reduced glutathione
TEM: Transmission electron microscopy
TNF-α: Tumor necrosis factor-alpha
VCO: Virgin coconut oil
XRD: X-ray diffraction spectroscopy
